# The Outside of the Platter

**Published:** 1899-11

**Authors:** 


### ARTICLE IX.

### "THE OUTSIDE OF THE PLATTER."

A few weeks ago we called attention in these pages to the prevalent custom, among cheap, advertising, and quack dentists, of using impression compound over and over, going from one mouth to another with it, without making any attempt at sterilization, until every particle of the mass must be thoroughly impregnated with nearly every form of micro-organism mentioned in the records of bacteriology. Furthermore, our attention has been called to the advertisements of several dental manufacturing companies, wherein they offer for sale a soft or "renovating" form of impression compo., for mixing with that which has been used for some time, to improve its plasticity. This certainly seems to indicate that the custom is a general one. Should this supposition prove to be well founded, we shall have a most glaring instance in a habit in our profession of that "cleansing of the outside of the platter," which is mentioned with much scorn in a certain wise old book. The illustration is quite complete, for in one of our most recently received exchanges the sterilization of impression cups is insisted on, but not a word is uttered concerning the sterilization of that which goes into the cup; nor do we recall ever having seen or heard a word written or spoken on this subject.

Is it true that while our journals for the last few years have contained hundreds of articles, giving minute instructions for the sterilization of excavators, scalers, burs, pluggers, forceps, and even engine hand pieces, that the one thing most likely to convey bacteria from one mouth to another has been entirely overlooked? Verily, this is even worse than the custom of the medical man who goes from mouth to mouth with his clinical thermometer. Are we such creatures of habit and fads that we never think, that it never occurs to us to do anything except whatever may be the fashion for the moment? There is material here for much serious reflection. If, as now seems probable, it should be demonstrated that the power of certain bacteria to convert carbohydrates into acid may, like other pathogenic qualities of micro-organisms which have become firmly fixed from extended action and inheritance, persist a considerable time even after change of environment, then may it not be possible to introduce caries-producing bacteria into a healthy mouth, or a more rapidly acting form of the micro-organisms which produce decay of the teeth into mouths where this disease has been progressing but slowly? And if, as is now believed by many—and as one investigator, at least, claims to have demonstrated—it should appear that pyorrhea alveolaris has its specific bacterium, may we not find in this very promiscuous use of impression compo., an explanation of the rapid increase of this baffling disorder? We hope to see our exchanges take this matter up at once and vigorously deal with it. No argument is needed in asking for immediate and concerted action for the discontinuance of a practice which must shock the sense of propriety of all decent people; and, what is of greater importance, must also be gravely dangerous to the health of our patients.—Editorial in The Dentist.

---

ABOUT PATHOLOGY.—What place is this? This is the Pathological Society. How does one know it is the Pathological Society? You know it by the specimens and smells.

What does that gentleman say? He says he has made a post-mortem. All the gentlemen make post-mortems. They would rather make post-mortems than go to a party.

What is that on a plate? That is a tumor. It is a very large tumor. It weighs one hundred and twelve pounds. The patient weighed eighty-eight pounds. Was the tumor removed from the patient? No, the patient was removed from the tumor. Did they save the patient? No, but they saved the tumor.

What is this in the bottle? It is tape-worm. It is a long tape worm; it is three-quarters of a mile long. Is that much for a tape-worm? It is indeed much for a tape-worm, but not much for the Pathological Society.—Indiana Medical Journal.

